# Characterization of Mitochondrial Alterations in Aicardi–Goutières Patients Mutated in *RNASEH2A* and *RNASEH2B* Genes

**DOI:** 10.3390/ijms232214482

**Published:** 2022-11-21

**Authors:** Francesca Dragoni, Jessica Garau, Daisy Sproviero, Simona Orcesi, Costanza Varesio, Silvia De Siervi, Stella Gagliardi, Cristina Cereda, Orietta Pansarasa

**Affiliations:** 1 Department of Biology and Biotechnology, University of Pavia, 27100 Pavia, Italy; 2 Molecular Biology and Transcriptomics Unit, IRCCS Mondino Foundation, 27100 Pavia, Italy; 3 Neurogenetics Research Centre, IRCCS Mondino Foundation, 27100 Pavia, Italy; 4 IFOM—The FIRC Institute of Molecular Oncology, 20139 Milan, Italy; 5 Department of Brain and Behavioral Sciences, University of Pavia, 27100 Pavia, Italy; 6 Department of Child Neurology and Psychiatry, IRCCS Mondino Foundation, 27100 Pavia, Italy; 7 Department of Women, Mothers and Neonatal Care, Children’s Hospital “V. Buzzi”, 20154 Milan, Italy; 8 Cellular Model and Neuroepigenetics Unit, IRCCS Mondino Foundation, 27100 Pavia, Italy

**Keywords:** Aicardi–Goutières syndrome, mtDNA, mitochondrial disorder, mitochondrial stress, ROS, IFN-α, inflammation, autoimmune diseases

## Abstract

Aicardi–Goutières syndrome (AGS) is a rare encephalopathy characterized by neurological and immunological features. Mitochondrial dysfunctions may lead to mitochondrial DNA (mtDNA) release and consequent immune system activation. We investigated the role of mitochondria and mtDNA in AGS pathogenesis by studying patients mutated in *RNASEH2B* and *RNASEH2A* genes. Lymphoblastoid cell lines (LCLs) from *RNASEH2A*- and *RNASEH2B*-mutated patients and healthy control were used. Transmission Electron Microscopy (TEM) and flow cytometry were used to assess morphological alterations, reactive oxygen species (ROS) production and mitochondrial membrane potential variations. Seahorse Analyzer was used to investigate metabolic alterations, and mtDNA oxidation and VDAC1 oligomerization were assessed by immunofluorescence. Western blot and RT-qPCR were used to quantify mtTFA protein and mtDNA release. Morphological alterations of mitochondria were observed in both mutated LCLs, and loss of physiological membrane potential was mainly identified in *RNASEH2A* LCLs. ROS production and 8-oxoGuanine levels were increased in *RNASEH2B* LCLs. Additionally, the VDAC1 signal was increased, suggesting a mitochondrial pore formation possibly determining mtDNA release. Indeed, higher cytoplasmic mtDNA levels were found in *RNASEH2B* LCLs. Metabolic alterations confirmed mitochondrial damage in both LCLs. Data highlighted mitochondrial alterations in AGS patients’ LCLs suggesting a pivotal role in AGS pathogenesis.

## 1. Introduction

Aicardi–Goutières syndrome (AGS) is an early-onset rare genetically determined encephalopathy, whose phenotype encompasses a wide range of neurological and extraneurological manifestations. From the neurological point of view, AGS patients usually demonstrate a congenital or sub-acute onset during their first year of life variably manifesting as microcephaly, spasticity, dystonia, seizures, cortical blindness and psychomotor delay. Radiological and laboratory assessments usually show intracranial calcification, white matter abnormalities, cerebral atrophy and cerebrospinal fluid lymphocytosis. Other extraneurological characteristics, such as chilblains, glaucoma, hypothyroidism, raised levels of autoantibodies and lupus-like disease, were also identified as persistent correlations with this disease [1]. Up to now, nine genes (*TREX1*, *RNASEH2B*, *RNASEH2A*, *RNASEH2C*, *ADAR1*, *SAMHD1*, *IFIH1*, *LSM11* and *RNU7*) associated with AGS phenotype with an autosomal recessive or dominant inheritance have been discovered, which are all involved in nucleic acids (NAs) metabolism or signaling [2,3,4]. Moreover, patients with AGS consistently present high levels of IFN-α activity in the cerebrospinal fluid and serum [5] and an increased expression of IFN-α-stimulated genes (ISGs) in peripheral blood, which is known as the interferon signature [6,7]. These findings are crucial to defining AGS as an inflammatory disease, characterized by the induction of a type I interferon-mediated innate immune response, which is most likely triggered by endogenously derived NAs. 

Emerging evidence has established a strong relationship between autoimmune diseases and mitochondrial dysfunctions [8,9]. In support of these considerations, several autoimmune disorders are associated with increased anti-mitochondrial antibodies and oxidative stress indicators, suggesting the proven function of mitochondria in the production of reactive oxygen species (ROS) must be considered [10].

Mitochondria are the primary energy-generating organelles in cells, producing adenosine triphosphate (ATP) via the tricarboxylic acid cycle (TCA) and oxidative phosphorylation (OXPHOS) at electron transport chain (ETC) complexes [8,11]. Mitochondria are also essential for heat production, calcium storage, reduction-oxidation (redox) regulation, biosynthesis, cell signaling and apoptosis, and recently, it was demonstrated that they have an important role in adaptive and innate immunity [11,12]. In particular, it was proven that the release of mitochondrial DNA (mtDNA) into the blood is increased in a variety of inflammatory disorders [13]. Defective mitochondria contribute to increased ROS levels and activate the intrinsic apoptotic cell death pathway, amplifying oxidative stress and leading to the release of mtDNA into the cytosol [14]. mtDNA interacts with and activates a wide variety of immunostimulatory DNA sensors, such as cyclic guanosine monophosphate-adenosine monophosphate synthase (cGAS) and Toll-like receptors (TLR7/9), which can induce autoimmunity, activating type I interferon inflammatory responses [15].

Differences in mitochondrial function are linked to dynamics in mitochondrial morphology. Changes in mitochondrial shape, size and localization, caused by mitochondrial dynamics events, such as fusion and fission, have been shown to have many effects in regulating T cell activity [16]. In addition, mitochondrial morphological changes, which are metabolically controlled, are also influenced by mitophagy, a critical process for mitochondrial turnover and for maintaining both cellular function and intracellular homeostasis [17].

Currently, no studies have been implemented to investigate the association between mitochondrial alteration and inflammation in AGS. *RNASEH2A*, *RNASEH2B* and *RNASEH2C* encode for the three distinct subunits of RNase H2 protein, and they are the most commonly mutated genes in AGS patients. In eukaryotes, this enzyme is the primary source of cellular ribonuclease activity, and it may play a role in numerous phases of nucleic acid metabolism. Thus, this work focused on the role of mitochondria in AGS, both from a functional and a morphological point of view in lymphoblastoid cell lines (LCLs) derived from AGS patients mutated in the *RNASEH2A* and *RNASEH2B* genes, to ultimately establish the role of mitochondria in the pathogenesis of AGS.

## 2. Results

### 2.1. Functional and Morphological Alteration in Mitochondria of RNASEH2B- and RNASEH2A-Mutated LCLs

**Figure 1:**
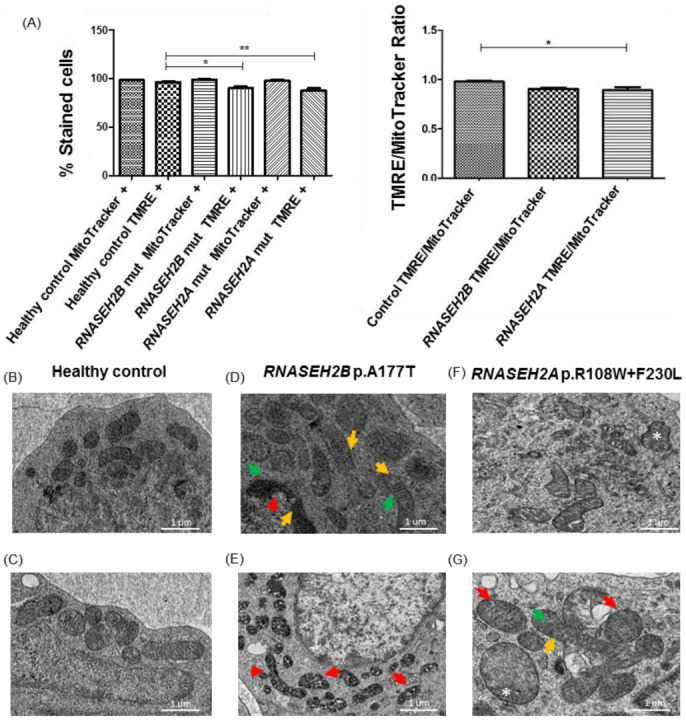
Mitochondria functionality and morphology of RNASEH2-mutated LCLs and healthy control. (**A**) Quantification of the TMRE and MitoTracker^TM^ signals and TMRE/Mitotracker Ratio from three independent experiments. MitoTracker^TM^ positive mitochondria represent the total number of mitochondria present in the cells. TMRE positive mitochondria represent the percentage of mitochondria with a not altered membrane potential in mutated *RNASEH2B* (p.A177T) and *RNASEH2A* (p.R108W + p.F230L) LCLs compared to the healthy control. The bar graph represents the mean of three biological experiments. ANOVA test and post hoc analysis Tukey’s Test have been performed. * *p* < 0.05, ** *p* < 0.01. (**B**–**G**) Representative TEM images of mitochondria from AGS patients carrying mutations in *RNASEH2B* (**D**,**E**) and *RNASEH2A* (**F**,**G**) genes show alterations of the physiological morphology. Mitochondria have unusual shapes (green arrows) (**D**,**G**) with packed cristae or overlaid layers of membranes (red arrows) (**D**,**E**,**G**) or with a longitudinal shape (yellow arrows) (**D**,**G**). Some mitochondria are enlarged and loss of cristae is observed (asterisk) (**F**,**G**).

Considering the difficulties in obtaining primary cells from children affected by a rare disease, in this study, we decided to use immortalized LCLs derived from one patient carrying mutation in the *RNASEH2B* (p.A177T) gene, one patient carrying mutation in the *RNASEH2A* (p.R108W + p.F230L) gene and one healthy control. Mitochondrial alterations and dysfunctions may lead to the release of mtDNA and, consequently, activate an IFN-α-mediated immune response [15]. To exert its functions properly, a healthy mitochondrion must maintain a negative membrane potential of −180 mV [18]. Variation in mitochondrial metabolism is caused by changes in the physiological intermembrane potential, which can lead both to oxidative stress and mtDNA release. Starting from these assumptions, in order to assess mitochondrial functionality, flow cytometry was used to compare the membrane potential of mitochondria in LCLs bearing mutations in *RNASEH2B* and *RNASEH2A* genes to that of the healthy control. MitoTracker^TM^ identifies the total number of mitochondria, while Tetramethylrhodamine Ethyl Ester (TMRE) marks only mitochondria which maintain their physiological potential and which are still functioning. In Figure 1A, we can see that both mutated LCLs show a higher percentage of mitochondria with an altered membrane potential (TMRE negative mitochondria) than the healthy control. The percentage of altered mitochondria appears to be significant in both mutated cell lines but, in particular, in the *RNASEH2A*-mutated cell lines. This result allowed us to hypothesize an impairment of these organelles in AGS patients. Functional impairment is often related to morphological alterations, responsible for mitochondrial dysfunction and mtDNA release. So, we decided to investigate the mitochondrial morphology in *RNASEH2B*- and *RNASEH2A*-mutated LCLs. TEM highlighted morphological alterations of mitochondria in both mutated LCLs compared to the healthy control (Figure 1B–G). Differently from the mitochondria of healthy control LCLs, which showed physiological structures, dimension and inner organization, the mitochondria of mutated LCLs exhibited unusual shapes (green arrows) (Figure 1D,G), with packed cristae or overlaid layers of membranes (red arrows) (Figure 1D,E,G); some of them seemed to be enlarged and showed loss of cristae (asterisk) (1F,G). We also observed that the mitochondria of *RNASEH2B*-mutated LCLs appear to be contained within spherical substructures containing fragments or sections of the damaged organelles, suggesting a more stressful condition for these patients (Figure 1E). These findings back up our hypothesis that mitochondrial damage could be linked to the onset of the disease, so in order to verify it, we decided to continue our research by studying mitochondrial stress.

### 2.2. Mitochondrial Stress and Metabolic Alteration in RNASEH2B- and RNASEH2A-Mutated LCLs

**Figure 2:**
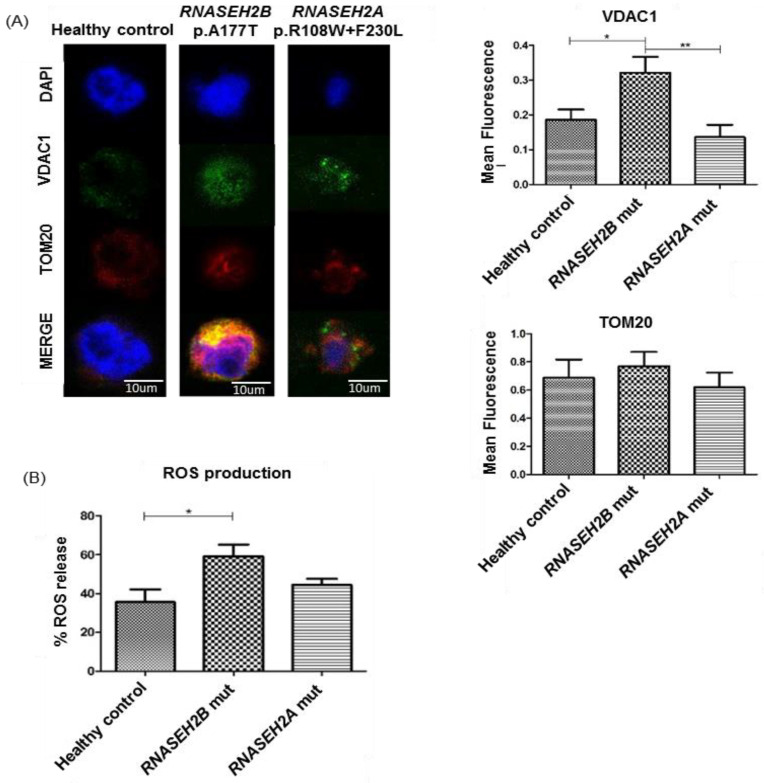

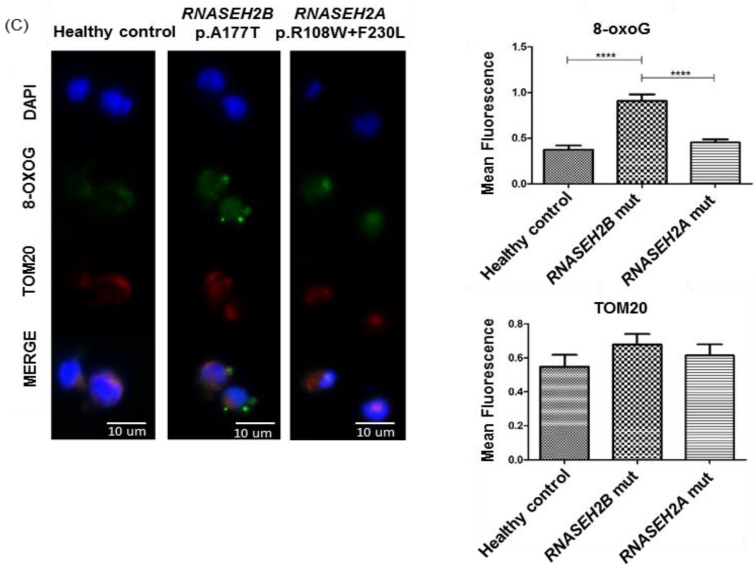
Mitochondrial stress in RNASEH2-mutated LCLs and healthy control. (**A**) Immunofluorescence and mean fluorescence (representative confocal images-Leica DM IRBE, Leica Microsystems Srl, Buccinasco, Italy) of LCLs derived from a healthy control and AGS patients mutated in *RNASEH2B* and *RNASEH2A* genes stained with VDAC1 antibody (green), TOM20 (red) and DAPI for nuclear staining (blue). ANOVA test and post hoc analysis Tukey’s Test have been performed. * *p* < 0.05, ** *p* < 0.01. (**B**) Flow cytometry analysis of ROS production in AGS patients mutated in *RNASEH2B* and *RNASEH2A* genes. The bar graph represents the mean of three biological experiments. ANOVA test and post hoc analysis Tukey’s Test have been performed. * *p* < 0.05. (**C**) Immunofluorescence and mean fluorescence of LCLs derived from a healthy control and AGS patients mutated in *RNASEH2B* and *RNASEH2A* genes stained with 8-oxoG antibody (green), TOM20 (red) and DAPI for nuclear staining (blue). ANOVA test and post hoc analysis Tukey’s Test have been performed. **** *p* < 0.0001.

Changes in membrane potential and mitochondrial stress cause oxidative stress and lead to an increased production of damaging ROS. The collapse of the membrane potential and the loss of physiological morphology, in particular, control the creation and the gating of a specific outer membrane pore, which might act as a release pathway for ROS and other residuum substrates under stressful conditions. The Voltage-Dependent Anion Channel 1 (VDAC1) aggregation is triggered by mitochondrial membrane potential and structural changes [15,19]; thus, we evaluated the VDAC1 signal level to study the pore organization by IF. We stained *RNASEH2B* and *RNASEH2A* LCLs with the monoclonal anti-VDAC1 antibody and the monoclonal translocase of the mitochondrial outer membrane (TOM20) antibody specific for the outer mitochondrial membrane (Figure 2A). Even though we previously reported a significantly higher percentage of mitochondria with altered membrane potential in *RNASEH2A*-mutated LCLs (Figure 1A), here, we observed increased signal of the VDAC1 in *RNASEH2B*-mutated LCLs, and notably, we found a marked colocalization between VDAC1 and TOM20 mainly in the *RNASEH2B*-mutated LCLs (Figure 2A, Figure S1). Moreover, we observed a different signal distribution between the two mutated lines: a more homogeneous distribution in *RNASEH2B*-mutated LCLs and a spot distribution in the *RNASEH2A*-mutated LCLs (Figure 2A). Since the VDAC1 oligomers form pores which could have a possible role in ROS release, we wondered if the production and the release of ROS in our LCLs could be increased. ROS production was investigated by flow cytometry using the cell permeant reagent 2′, 7′-Dichlorofluorescin Diacetate (DCFDA) to quantitatively assess ROS in live cell samples. According to our previous results (Figure 2A), we found that the *RNASEH2B*-mutated LCLs showed a higher level of ROS production when compared to the *RNASEH2A*-mutated LCLs and to the healthy control (Figure 2B). ROS are extremely reactive chemicals that can destroy lipids, proteins and nucleic acids. Guanine is the nucleobase that is most sensitive to ROS oxidation, resulting in the formation of 8-oxoguanine (8-oxoG) [20]. mtDNA contains bacteria-like unmethylated CpG motifs which are the substrate of ROS oxidation [21]. From this assumption, we tried to explore if the increased ROS production could also be associated with an increased level of DNA oxidation. We analyzed 8-oxoG levels by IF using a specific monoclonal anti-8-oxoG antibody and the anti-TOM20 antibody (Figure 2C). We found that, also in this case, the 8-oxoG signal is increased in both mutated LCLs compared to the healthy control, although statistical significance is reached only in the *RNASEH2B*-mutated LCLs, and in the *RNASEH2A*-mutated LCLs, we detect only a slight increase. No colocalization was detected between 8-oxoG and TOM20 signals in both healthy control and mutated LCLs (Figure S2).

**Figure 3:**
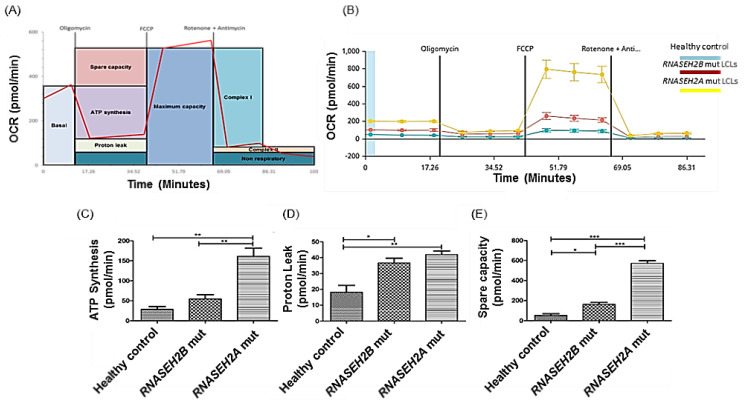

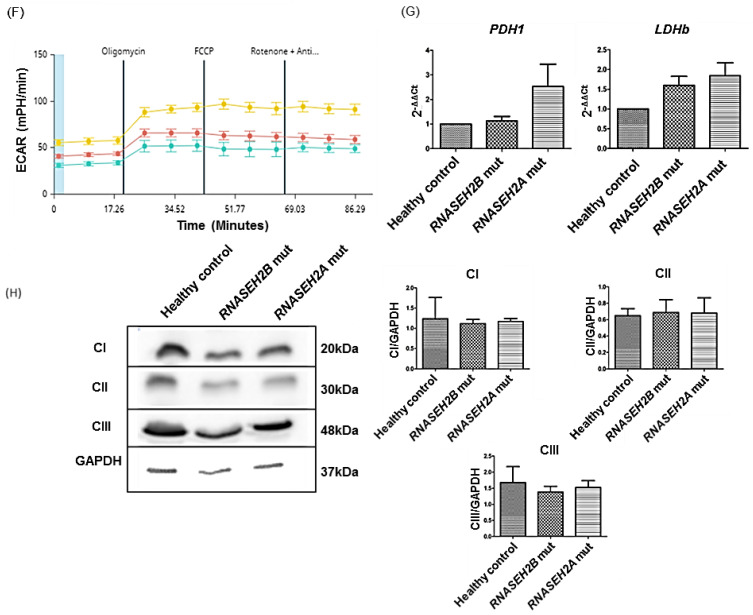
Metabolic and energetic status of LCLs derived from *RNASEH2B*- and *RNASEH2A*-mutated AGS patients. (**A**) Oxygen Consumption Rate (OCR in pmol/min) was measured using a Seahorse XFe24 Analyzer. OCR was measured in three independent experiments before (basal respiration) and after the addition of Oligomycin, FCCP and Rotenone/Antimycin A, allowing the determination of each component of the respiration chain. (**B**) OCR (pmol/min) of a representative experiment with standard errors reflecting the between-well variance within the experiment. Healthy control was indicated by the blue line, *RNASEH2B*-mutated LCLs by the red line and *RNASEH2A*-mutated LCLs by the yellow line. Three biological independent experiments have been performed. Data were collected and averaged from five separate wells for each cell line. (**C**) ATP synthesis, (**D**) Proton leak, (**E**) Spare respiratory capacity. ANOVA test and post hoc analysis Tukey’s Test have been performed. * *p* < 0.05, ** *p* < 0.01, *** *p* < 0.001. (**F**) Energetic status of LCLs derived from mutated AGS patients and healthy control. The Extracellular Acidification Rate (ECAR in pmol/min) was measured using a Seahorse XFe24 Analyzer. Healthy control was indicated by the blue line, *RNASEH2B*-mutated LCLs by the red line and *RNASEH2A*-mutated LCLs by the yellow line. Three biological independent experiments have been performed. Data were collected and averaged from five separate wells for each cell line. (**G**) mRNA expression levels of two glycolysis-related genes in AGS mutated LCLs and healthy control. Glycolysis-related genes, pyruvate dehydrogenase kinase 1 (*PDK1*) and Lactate dehydrogenase B (*LDHb*) were quantified using qRT-PCR on mutated LCLs and healthy control. *GAPDH* was used to normalize the gene expression. The bar graph represents the mean of three biological experiments. ANOVA test and post hoc analysis Tukey’s Test have been performed. (**H**) OXPHOS system complexes. Representative Western blot and histogram of OXPHOS system complexes protein levels in LCLs derived from a healthy control and AGS patients. The *GAPDH* protein level was used to normalize each OXPHOS system complex.

In addition, mitochondrial activities and physiology are strictly related to oxidative phosphorylation and Electron Transport Chain (ETC). Impaired or higher mitochondrial electron transport rates of apparently normal mitochondrial respiration can also result in increased ROS production [22,23]. We wondered if mitochondrial dysfunctions in AGS patients’ cell lines could also involve metabolic and bioenergetics characteristics. We used the Seahorse Analysis to assess mitochondrial functionality by directly measuring the Oxygen Consumption Rate (OCR) in live cells. We injected three compounds which act as modulators of specific steps of the ETC: Oligomycin acts on the ATP Synthase (complex V), Carbonyl cyanide-4 (trifluoromethoxy) phenylhydrazone (FCCP) acts on the inner mitochondrial membrane and Rotenone/Antimycin A exerts its action on the Complex I and Complex III (Figure 3A,B). Our findings revealed that mitochondrial respiration and ATP production rates are substantially increased in AGS mutated LCLs compared to the healthy control (Figure 3C). As a result of the increased mitochondrial transport rate in AGS patients, ROS production is also higher, as highlighted by the increased level of proton leak in mutated LCLs (Figure 3D), thus confirming our previous findings. We further looked at the energetic status of our cell lines by using the Seahorse Analysis to directly measure the Extracellular Acidification Rate (ECAR), which demonstrated that the glycolytic activity was increased in the *RNASEH2B* and, notably, in *RNASEH2A*-mutated LCLs (Figure 3F). The increased recovery capability after mitochondrial damage is highlighted by the increased spare capacity in the *RNASEH2A*-mutated LCLs (Figure 3E) which hold the catalytic function of the complex. The increased glycolytic activity in *RNASEH2A*-mutated LCLs was verified by qRT-PCR (Figure 3G). Conversely, Western blot analysis of the OXPHOS System Complexes revealed no significant changes between mutated LCLs and the healthy control (Figure 3H).

### 2.3. mtDNA Release from Altered Mitochondria of RNASEH2B- and RNASEH2A-Mutated LCLs

**Figure 4:**
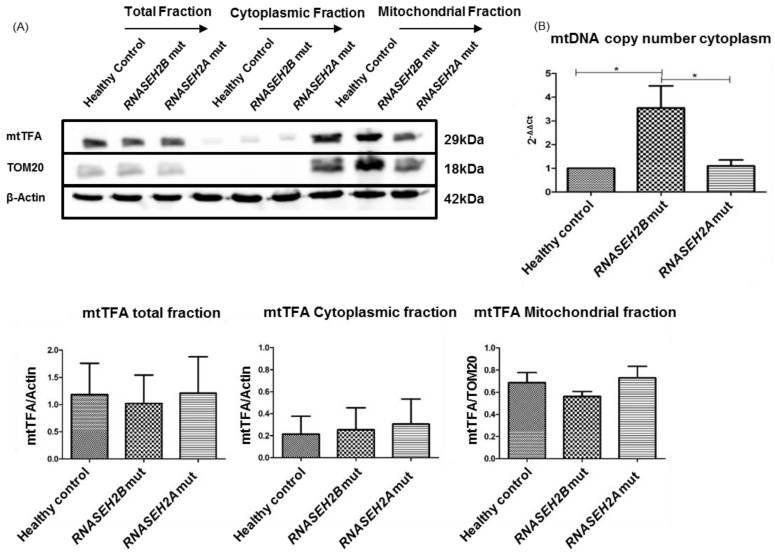
Correlation between mtTFA protein level and mtDNA release from altered mitochondria in *RNASEH2B*- and *RNASEH2A*-mutated LCLs. (**A**) Representative Western blot and histogram of the mtTFA protein level in LCLs derived from healthy control and AGS patients. Β-Actin was used to normalize the total and the isolated cytoplasmic fraction and TOM20 was used to normalize the isolated mitochondrial fraction of the proteins. (**B**) Expression of mitochondrial *MT-TL1* in healthy control and AGS patients (*RNASEH2B*- and *RNASEH2A*-mutated). *B2M* was used to normalize the gene expression. The bar graph represents the mean of three biological experiments. ANOVA test and post hoc analysis Tukey’s Test have been performed. * *p* < 0.05.

Morphological and functional alterations of mitochondria are often related to mtDNA release from damaged organelles [19,24]. mtDNA cytoplasmic accumulation could activate an abnormal IFN-α-mediated immune response and lead to the upregulation of ISGs. Considering that our previous results highlighted a mitochondrial impairment, we wondered if mtDNA release could also be involved in AGS. As reported in the literature [24,25], the mitochondrial transcription factor A (mtTFA) is responsible for replication, packaging and transcriptional regulation of mtDNA. Western blot analysis was used to assess mtTFA protein level and, as described in Figure 4A, a reduction of this protein in total and mitochondrial fractions of mutated LCLs compared to the healthy control was observed, especially in *RNASEH2B*-mutated LCLs. Since there is a correlation between mtTFA protein level and the release of mtDNA from injured mitochondria, we also tried to determine the number of mtDNA copies in LCLs. Assuming that mtTFA reduction is associated with the release of mtDNA from damaged organelles, we isolated the cytoplasmic compartment to demonstrate the plausible ectopic presence of mitochondrial NAs. Accordingly, qRT-PCR showed a significant increase in the mtDNA copy number in *RNASEH2B* LCLs’ cytoplasm, confirming the potential mitochondrial disruption and the consequent release of mtDNA into the cytoplasm as a possible trigger of IFN-α-mediated immune response (Figure 4B). 

## 3. Discussion

Aicardi–Goutières syndrome (AGS) is a rare, genetically determined encephalopathy, with typical onset within the first year of life, and is inherited in autosomal recessive and dominant form. All AGS-related genes encode for proteins implicated in the DNA damage response and NAs metabolism, and their mutations may cause an incorrect innate immune response, resulting in elevated IFN-α production [2,26]. *RNASEH2A*, *RNASEH2B* and *RNASEH2C* encode for the three distinct subunits of RNAse H2 protein, and they are the most commonly altered genes in AGS patients [2,3,27]. In eukaryotes, this enzyme is the primary source of cellular ribonuclease activity, and it may play a role in numerous areas of NAs metabolism [28]. Several studies have demonstrated the involvement of mitochondrial impairment in autoimmune and inflammatory diseases, with a strong correlation between the mtDNA release and the induction of an abnormal IFN-α dependent immune response [29,30]. Up to now, no studies have been performed to directly relate the AGS pathogenesis with mitochondrial alterations; therefore, in our work, we tried to investigate from a morphological and functional point of view this association. We found that both mutated *RNASEH2B* and *RNASEH2A* LCLs derived from AGS patients showed an alteration of the physiological negative intermembrane potential, with a significant increase in damaged mitochondria in the *RNASEH2A*-mutated LCLs. As reported in the literature, alterations of the membrane potential [18] could be a signal of mitochondrial dysfunction and it has also been described in Systemic Lupus Erythematosus (SLE) [31], a disease which shares many features with AGS. This leads us to hypothesize a potential functional and morphological stress in these organelles of AGS patients. We went ahead investigating the morphological characteristics of these subcellular elements and, not surprisingly, TEM analysis confirmed the mitochondrial structural impairment. The mitochondria of both mutated LCLs showed altered organization, size and shapes. Lots of them exhibited loss of cristae and modified structures, with the presence of substructures containing parts of damaged organelles, especially in *RNASEH2B*-mutated cell line. *RNASEH2B*-mutated LCLs also showed a greater stressful condition when compared to the other mutant and the healthy control, as reported in Garau et al., 2021 [32]. Morphological and functional alterations are often related to oxidative stress and ROS production increase in neurodegenerative and inflammatory disorders, including SLE [10,33,34]. The increased signal of the VDAC1 protein in both mutated LCLs, and especially in the cell line mutated in *RNASEH2B*, could indicate that this channel is involved in the release of ROS [19] in response to structural changes. We also identified a distinct signal distribution of the VDAC1. The oligomerized form of this pore could be represented by the homogeneous distribution and strong colocalization with the TOM20 signal in *RNASEH2B*-mutated LCLs, as opposed to the point distribution and not-colocalized signal with TOM20 in *RNASEH2A*-mutated LCLs. Here, it could represent the non-oligomerized form of the pore and be related to the least stressful condition for this cell line. Accordingly, the evaluation of ROS levels confirmed a higher percentage of ROS production in the *RNASEH2B*-mutated cell line. It is reported in the literature that ROS could have an impact on the oxidation of several substrates and especially on the nucleobase guanine, with the consequent production of 8-oxoguanine [20,21,35], which in turn could induce the inflammatory response. Additionally, in this case, our findings supported our hypothesis and we found that, when compared to healthy control, the 8-oxoG signal is stronger in both altered LCLs, but mainly in the *RNASEH2B*-mutated LCLs. In both healthy control and mutated LCLs, no colocalization of 8-oxoG and TOM20 signals was identified, but the enhanced signal of oxidized DNA in *RNASEH2B*-mutated LCLs due to ROS generation from damaged mitochondria prompted us to speculate a potential influence on genomic DNA. Moreover, considering that mitochondrial physiology and activities are inextricably linked to oxidative phosphorylation and the ETC [22,36], we assess the metabolic and bioenergetic status of each cell line. According to our previous findings, mitochondrial respiration and ATP generation rates seem to be substantially higher in AGS mutant LCLs than in the healthy control. ROS production is higher in AGS patients as a result of the increased mitochondrial transport rates, as seen by the increased level of proton leak in mutant LCLs, corroborating our previous findings. We also looked at the energy condition of our cell lines and discovered that mutated LCLs had higher glycolytic activity than healthy control, especially in *RNASEH2A* mutant LCLs. We hypothesized that the catalytic subunit of RNAseH2 complex, which must maintain its activity even under stress, may try to boost its energetic status by increasing ATP synthesis through the glycolytic pathway. These results were also corroborated by the increased expression levels, in the *RNASEH2A*-mutant LCLs, of two glycolytic genes (*LDHb* and *PDK1*). In contrast, protein levels in the OXPHOS system complexes did not show any meaningful difference, indicating that these complexes are not more expressed in response to stress conditions but are possibly more active. The release of mtDNA into the blood has been found to be elevated in a range of inflammatory illnesses in response to mitochondrial dysfunction. Defective mitochondria increase ROS levels and trigger the intrinsic apoptotic cell death pathway, incrementing oxidative stress and allowing mtDNA to leak into the cytoplasm. There, mtDNA interacts with and activates a variety of immunostimulatory DNA sensors, including cyclic guanosine monophosphate-adenosine monophosphate synthase (cGAS) and Toll-like receptors (TLR7/9), which can trigger autoimmunity by activating IFN-α inflammatory responses [13,15,31]. We previously established the activation of immunological pathways downstream of TLR7/9 as well as the activation of ISGs (*IFI44* and *IFIT1*) in Garau et al., 2021 [32]. Different forms of NAs, including mtDNA, can trigger an immunological response and, as a result, induce the production of IFN-α, which is one of the main characteristics in AGS patients. A similar mechanism has also been described in association with SLE where the mtDNA released in the cytoplasm may trigger antiviral signaling, leading in turn to inflammation [37]. We found a reduction in the mtTFA protein level in mutated LCLs, especially in *RNASEH2B* LCLs, which is related to the possible release of mtDNA from damaged mitochondria. Accordingly, the mtDNA copy number in *RNASEH2B*-mutated LCLs was increased, suggesting the effective possible release of this NA into the cytoplasm, which could be responsible for the activation of an IFN-α-mediated immune response [30].

In conclusion, our findings showed that mitochondrial dysfunctions might play a critical role in AGS pathogenesis. We speculated that AGS patients, with different mutations, may have distinct responses to mitochondrial stress. Through improved energy metabolism, the *RNASEH2A* mutant cell line demonstrated higher recovery capability following oxidative stress damage. The patient with the *RNASEH2B* mutation had a more severe stress response, with greater levels of ROS production and mtDNA release. We further speculated that mtDNA release could be responsible for the activation of many immunological pathways, including the cGAS-STING and TLR7/9 pathways, as we described in one of our previous works [32].

## 4. Materials and Methods

### 4.1. Patients’ Enrolment

The IRCCS Mondino Foundation in Pavia, Italy, enrolled AGS patients. One 9-year-old male patient carrying a homozygous mutation in the *RNASEH2B* (p.A177T) gene and one 13-year-old female patient carrying a compound heterozygous mutation in the *RNASEH2A* (p.R108W + p.F230L) gene were recruited. Patients were diagnosed based on clinical advice. To confirm the pathology, genetic analysis has been previously performed in our laboratory [3]. A healthy volunteer (female, age 23) was recruited at the IRCCS Policlinico S. Matteo Foundation’s Immunohematological and Transfusional Service in Pavia, Italy, who was free of any pharmacological therapy or pathology. All samples used in the experiments were taken with the agreement of the subjects. 

### 4.2. Cells Isolation

Following the manufacturer’s instructions, peripheral blood mononuclear cells (PBMCs) were separated from peripheral venous blood using Histopaque^®^-1077 (Sigma-Aldrich, St. Louis, MO, USA). Blood samples were placed in a centrifuge tube with an equal volume of Histopaque^®^-1077 and spun at 300 g with low deceleration for 30 min. The PBMCs were then washed in 1X PBS after being retrieved from the intermediate phase (Sigma-Aldrich, St. Louis, MO, USA). The cells were centrifuged for 10 min at 200× *g* and the supernatant was discarded.

### 4.3. EBV-Immortalization and Cell Culture

Doctor Chiara Baldo of the Laboratorio di Genetica Umana, IRCCS Istituto Giannina Gaslini, Genoa, performed the EBV immortalization. LCLs with mutations in the *RNASEH2A* (p.R108W + p.F230L) and *RNASEH2B* (p.A177T) genes were studied, as well as one healthy control (CTRL). Cell lines were grown in RPMI 1640 medium (CARLO ERBA Reagents S.r.l., Cornaredo, Italy), supplemented with 20% fetal bovine serum (FBS) (CARLO ERBA Reagents S.r.l., Cornaredo, Italy), 0.3 mg/L L-glutamine, and 5% penicillin-streptomycin (CARLO ERBA Reagents S.r.l., Cornaredo, Italy) at 37 °C in a humidified atmosphere with 5% of CO_2_. Centrifugation was used to pellet cells, which were then washed in 1X PBS and treated as needed. 

### 4.4. Transmission Electron Microscopy (TEM) Analysis

Approximately 3 × 10^6^ living cells were rinsed in 1X PBS and treated for 4 h at 4 °C with the fixing solution (2.5% glutaraldehyde and 2% paraformaldehyde in cacodylate buffer, pH 7.3), then post-fixed in 1.5% osmium tetroxide for 1 h at room temperature (RT) and Epon-Araldite embedding. Mr. Raffaele Allevi of the Department of Biomedical and Clinical Sciences “L. Sacco,” in Milano obtained the ultrathin slices (~70 nm thick) from the resin blocks and stained them with uranyl acetate/lead citrate before being observed using conventional transmission electron microscopy (TEM) [38].

### 4.5. TMRE Flow Cytometry Analysis 

About 1 × 10^5^ living cells were resuspended in a staining mix composed of 1X PBS, 0.2% BSA with the addition of 200 nM of Mitotracker Green (Thermo Fisher Scientific, Waltham, MA, USA) and 200 nM of TMRE (Abcam, Cambridge, UK). Successively, cells were incubated for 20 min at 37 °C, followed by centrifugation at 600× *g* for 5 min. After centrifugation, the supernatant was discarded, and the pellet was resuspended in 300 μL of 1X PBS. Finally, the cell suspension was analyzed by flow cytometer BD FACSCanto II, and raw data were analyzed with BD FACSDiva software (BD Biosciences, Franklin Lakes, NJ, USA).

### 4.6. ROS Production Flow Cytometry Analysis

ROS production was analyzed using the DCFDA/H2DCFDA-Cellular ROS Assay Kit (ab113851, Abcam, Cambridge, UK) according to manufacturer’s instructions. About 1 × 10^5^ living cells were collected and stained with cell-permeant reagent 2′,7′- dichlorofluorescein (DCFDA) for 30 min at 37 °C. Cells were then centrifuged at 1600× *g* for 8 min and the pellet was resuspended in 300 μL of 1X PBS. The cell suspension was analyzed by flow cytometer BD FACS Canto II, and raw data were analyzed with BD FACSDiva software (BD Biosciences, Franklin Lakes, NJ, USA).

### 4.7. Immunofluorescence (IF)

About 1 × 10^5^ living cells were placed on a poly-L-Lysine slide (Thermo Fisher Scientific, Waltham, MA, USA) and incubated at RT for 30 min. Cells were then fixed using a solution of 4% paraformaldehyde for 15 min at RT. Fixed cells were then permeabilized with 0.1% Triton™ X-100 (Sigma-Aldrich, St. Louis, MO, USA) for 10 min. Samples were treated with a blocking solution (0.05% Triton™ X-100, 1% BSA in 1X PBS) and then were incubated with the primary antibody for 2 h at RT and incubated with secondary antibody for 1 h at RT. They were finally washed with 1X PBS, mounted with ProLong^®^ Gold Antifade Reagent with DAPI (Thermo Fisher Scientific, Waltham, MA, USA), dried and nail polished. Slides were analyzed with a confocal laser microscope using z-stack acquisition (Leica DM IRBE, Leica Microsystems Srl, Buccinasco, Italy). The following antibodies were used for immunofluorescence: monoclonal anti-VDAC1 antibody (sc-390996, Santa Cruz Biotechnology, Dallas, TX, USA, dilution 1:250), rabbit monoclonal anti-TOM20 antibody (#42406, Cell Signaling Technology, Danvers, MA, USA, dilution 1:250) and mouse monoclonal anti-8oxoG antibody (sc-130914, Santa Cruz Biotechnology, Dallas, TX, USA, dilution 1:250).

The corrected total cell fluorescence analysis (CTFC) was calculated according to described protocols using the following formula: CTCF = Integrated Density—(Area of selected cell X Mean fluorescence of background readings). 

### 4.8. Seahorse Analysis

Mitochondrial metabolism was studied using the Seahorse Cell Mito Stress Test kit (Agilent Technologies, Inc, Santa Clara, CA, USA), starting from 2.8 × 10^5^ cells and following manufacturers’ instructions. The analysis was performed with the Seahorse XFe24 Analyzer (Agilent Technologies, Inc, Santa Clara, CA, USA) which measures the Oxygen Consumption Rate (OCR) by the addition of the complex I inhibitor Rotenone (0.5 µM), the coupling efficiency by the addition of Oligomycin (1.5 µM), an ATP synthase inhibitor, and the Spare Respiratory Capacity (i.e., the ability of the energy substrate supply and oxidative phosphorylation to face increased energy demand) by the addition of FCCP (1 µM), which uncouples oxidative phosphorylation from ATP synthesis.

### 4.9. RIPA Proteins Extraction and Quantification

The extraction of soluble protein samples from 5 × 10^6^ cells was done with RIPA buffer (50 mM Tris-HCl [pH 8.0], 150 mM NaCl, 1% NP-40 and 12 mM Deoxycholic acid, supplemented with protease inhibitors). BCA technique (Sigma-Aldrich, St. Louis, MO, USA) and bovine serum albumin (BSA) (Sigma-Aldrich, St. Louis, MO, USA) were used to quantify protein concentration using NanoDrop (Thermo Fisher Scientific, Waltham, MA, USA).

### 4.10. Cytoplasmic and Mitochondrial Protein Extraction and Quantification

The extraction of soluble protein samples from mitochondrial and cytoplasmic fraction of 15 × 10^6^ living cells was performed using Mitochondria Extraction Buffer mix (MEB) (Abcam, Cambridge, UK) and Cytosol Extraction Buffer mix (CEB) (Abcam, Cambridge, UK), respectively, supplemented with protease inhibitors and Dithiothreitol (DTT). Cells were collected, centrifuged at 1600× *g* for 8 min and resuspended in 200 μL of CEB. Cell suspension was then incubated on ice for 10 min, pass through a needle and centrifuged at 700× *g* for 10 min at 4 °C. The pellet, which represents the mitochondrial fraction, was resuspended in 40 μL of MEB while the supernatant was centrifuged at 10,000× *g* for 30 min at 4 °C to obtain the cytosolic fraction. BCA technique (Sigma-Aldrich, St. Louis, MO, USA) and bovine serum albumin (BSA) (Sigma-Aldrich, St. Louis, MO, USA) were used to quantify protein concentration with NanoDrop (Thermo Fisher Scientific, Waltham, MA, USA).

### 4.11. Western Blot Analysis

Western blotting analysis was performed by SDS–polyacrylamide gel electrophoresis (SDS-PAGE). Samples containing 30 μg of proteins were loaded into 12.5% SDS-PAGE gel. Then, samples were transferred to PVDF membranes using a semi dry transfer apparatus (Trans-blot Turbo, Bio-Rad Laboratories, Hercules, CA, USA). Membranes were treated with a blocking solution, containing 5% of non-fat dry milk in 1X TBS-T buffer (10 mM Tris-HCl, 100 mM NaCl, 0.1% Tween, pH 7.5), for 1 h and then incubated overnight with primary antibodies at 4 °C. Immunoreactivity was detected using the donkey anti-rabbit or anti-mouse secondary peroxidase-conjugated (GE Healthcare, Chicago, IL, USA). The immunoreactive bands were visualized using the enhanced chemiluminescence detection kit (ECL Advance, GE Healthcare, Chicago, IL, USA). The following antibodies were used for Western blot analysis: mouse monoclonal anti-mtTFA (sc-376672, Santa Cruz Biotechnology, Dallas, TX, USA, dilution 1:500), mouse monoclonal anti-β-Actin (66009-1-Ig, Proteintech, Rosemont, IL, USA, dilution 1:10,000), rabbit monoclonal anti-TOM20 (#42406, Cell Signaling Technology, Danvers, MA, USA, Rosemont, IL, USA, dilution 1:1000), Total OXPHOS Rodent WB Antibody Cocktail (ab110413, Abcam, Cambridge, UK, dilution 1:500) and rabbit polyclonal anti-GAPDH (GTX100118, GeneTex, Irvine, CA, USA, dilution 1:10,000).

### 4.12. mtDNA Extraction 

Cytoplasmic mtDNA extraction from 1 × 10^6^ living cells was performed following Bronner and O’Riordan’s protocol [39], and using DNeasy Blood & Tissue kit (QIAGEN, catalog number:69504, Hilden, Germany) following manufacturers’ specifications. 

### 4.13. RNA Extraction with Trizol Reagent

RNA from LCLs was isolated with Trizol^®^ reagent (Sigma-Aldrich, St. Louis, MO, USA) according to manufacturer’s specifications. RNA was then quantified by NanoDrop ND1000 UV-Vis Spectrophotometer (Thermo Fisher Scientific, Waltham, MA, USA).

### 4.14. Reverse Transcription

RNA (800 ng) was reverse transcribed using the iScript™ Reverse Transcription Supermix kit for RT-qPCR (Bio-Rad Laboratories, Hercules, CA, USA), according to the manufacturer’s recommendations.

### 4.15. Real-Time PCR

For the mtDNA copy number quantification, the *MT*-*TL1* gene was analyzed. qPCR reactions included 200 nM of each oligonucleotide, 7.5 μL of SYBR Green SuperMix (BioRad, Richmond, CA, USA), and 1 μL of DNA template (50 ng/µL) or water control. 

For *PDK1* and *LDHb* analysis qPCR reactions included 200 nM of each oligonucleotide, 7.5 μL of SYBR Green SuperMix (BioRad, Richmond, CA, USA), and 1 μL of cDNA template or water control. Primers are indicated in Table 1. Cycle threshold (Ct) values were automatically recorded for each replicate qPCR reaction, and mean Ct values were normalized against those determined for the *B2M* gene for mtDNA and *GAPDH* gene for the cDNA. Fold-expression differences relative to healthy controls were determined using the 2^−ΔΔCt^ method.

### 4.16. Statistical Analysis

Means and standard deviations were used to represent the data. One-way Analysis of Variance (ANOVA) was used for statistical analysis, using Tukey’s test as a post hoc test (GraphPad Prism version 5, San Diego, CA, USA). When the *p*-values were less than 0.05, the results were considered statistically significant.

## Figures and Tables

**Table 1:** Primers sequences.

Gene Symbol	Forward Sequence	Reverse Sequence
*MT-TL1*	CACCCAAGAACAGGGTTTGT	TGGCCATGGGTATGTTGTTA
*B2M*	CAAATTCAAACCCAGCCTGT	TCCTGCCTGGAACTCTCTGT
*PDK1*	GAACACCATGCCAACAGAGG	TACCCAGCGTGACATGAACT
*LDHb*	CTTTGTCTTCTCCGCACGAC	CTCTTCTTCCGCAACTGGTG

## Data Availability

The obtained data are deposited on the Zenodo repository (DOI:10.5281/zenodo.5718535).

## Supplementary Materials

The following supporting information can be downloaded at: https://www.mdpi.com/article/10.3390/ijms232214482/s1.

## References

[1] Crow Y.J., Shetty J., Livingston J.H. (2020). Treatments in Aicardi-Goutières syndrome. Dev. Med. Child Neurol..

[2] Crow Y.J., Chase D.S., Schmidt J.L., Szynkiewicz M., Forte G.M., Gornall H.L., Oojageer A., Anderson B., Pizzino A., Helman G. (2015). Characterization of human disease phenotypes associated with mutations in TREX1, RNASEH2A, RNASEH2B, RNASEH2C, SAMHD1, ADAR, and IFIH1. Am. J. Med. Genet. A.

[3] Garau J., Cavallera V., Valente M., Tonduti D., Sproviero D., Zucca S., Battaglia D., Battini R., Bertini E., Cappanera S. (2019). Molecular Genetics and Interferon Signature in the Italian Aicardi Goutières Syndrome Cohort: Report of 12 New Cases and Literature Review. J. Clin. Med..

[4] Uggenti C., Lepelley A., Depp M., Badrock A.P., Rodero M.P., El-Daher M.T., Rice G.I., Dhir S., Wheeler A.P., Dhir A. (2020). cGAS-mediated induction of type I interferon due to inborn errors of histone pre-mRNA processing. Nat. Genet..

[5] Goutières F. (2005). Aicardi-Goutières syndrome. Brain Dev..

[6] Baechler E.C., Batliwalla F.M., Karypis G., Gaffney P.M., Ortmann W.A., Espe K.J., Shark K.B., Grande W.J., Hughes K.M., Kapur V. (2003). Interferon-inducible gene expression signature in peripheral blood cells of patients with severe lupus. Proc. Natl. Acad. Sci. USA.

[7] Bennett L., Palucka A.K., Arce E., Cantrell V., Borvak J., Banchereau J., Pascual V. (2003). Interferon and granulopoiesis signatures in systemic lupus erythematosus blood. J. Exp. Med..

[8] Takeshima Y., Iwasaki Y., Fujio K., Yamamoto K. (2019). Metabolism as a key regulator in the pathogenesis of systemic lupus erythematosus. Semin. Arthritis Rheum..

[9] Faas M.M., de Vos P. (2020). Mitochondrial function in immune cells in health and disease. Biochim. Biophys. Acta Mol. Basis Dis..

[10] Barrera M.-J., Aguilera S., Castro I., Carvajal P., Jara D., Molina C., González S., González M.-J. (2021). Dysfunctional mitochondria as critical players in the inflammation of autoimmune diseases: Potential role in Sjögren’s syndrome. Autoimmun. Rev..

[11] Meyer A., Laverny G., Bernardi L., Charles A.L., Alsaleh G., Pottecher J., Sibilia J., Geny B. (2018). Mitochondria: An Organelle of Bacterial Origin Controlling Inflammation. Front. Immunol..

[12] Rongvaux A. (2018). Innate immunity and tolerance toward mitochondria. Mitochondrion.

[13] Riley J.S., Tait S.W. (2020). Mitochondrial DNA in inflammation and immunity. EMBO Rep..

[14] Zhang X., Wu X., Hu Q., Wu J., Wang G., Hong Z., Ren J. (2019). Mitochondrial DNA in liver inflammation and oxidative stress. Life Sci..

[15] Kim J., Gupta R., Blanco L.P., Yang S., Shteinfer-Kuzmine A., Wang K., Zhu J., Yoon H.E., Wang X., Kerkhofs M. (2019). VDAC oligomers form mitochondrial pores to release mtDNA fragments and promote lupus-like disease. Science.

[16] Liesa M., Shirihai O.S. (2016). Mitochondrial Networking in T Cell Memory. Cell.

[17] Mohanty A., Tiwari-Pandey R., Pandey N.R. (2019). Mitochondria: The indispensable players in innate immunity and guardians of the inflammatory response. J. Cell Commun. Signal..

[18] Crowley L.C., Christensen M.E., Waterhouse N.J. (2016). Measuring mitochondrial transmembrane potential by TMRE staining. Cold Spring Harb. Protoc..

[19] Yan J., Liu W., Feng F., Chen L. (2020). VDAC oligomer pores: A mechanism in disease triggered by mtDNA release. Cell Biol. Int..

[20] Leon J., Sakumi K., Castillo E., Sheng Z., Oka S., Nakabeppu Y. (2016). 8-Oxoguanine accumulation in mitochondrial DNA causes mitochondrial dysfunction and impairs neuritogenesis in cultured adult mouse cortical neurons under oxidative conditions. Sci. Rep..

[21] Caielli S., Athale S., Domic B., Murat E., Chandra M., Banchereau R., Baisch J., Phelps K., Clayton S., Gong M. (2016). Oxidized mitochondrial nucleoids released by neutrophils drive type I interferon production in human lupus. J. Exp. Med..

[22] Annesley S.J., Lay S.T., De Piazza S.W., Sanislav O., Hammersley E., Allan C.Y., Francione L.M., Bui M.Q., Chen Z.P., Ngoei K.R.W. (2016). Immortalized Parkinson’s disease lymphocytes have enhanced mitochondrial respiratory activity. Dis. Models Mech..

[23] Indo H.P., Davidson M., Yen H.-C., Suenaga S., Tomita K., Nishii T., Higuchi M., Koga Y., Ozawa T., Majima H.J. (2007). Evidence of ROS generation by mitochondria in cells with impaired electron transport chain and mitochondrial DNA damage. Mitochondrion.

[24] Kang I., Chu C.T., Kaufman B.A. (2018). The mitochondrial transcription factor TFAM in neurodegeneration: Emerging evidence and mechanisms. FEBS Lett..

[25] West A.P., Khoury-Hanold W., Staron M., Tal M.C., Pineda C.M., Lang S.M., Bestwick M., Duguay B.A., Raimundo N., MacDuff D.A. (2015). Mitochondrial DNA stress primes the antiviral innate immune response. Nature.

[26] Lanzi G., Fazzi E., D’Arrigo S. (2002). Aicardi-Goutières syndrome: A description of 21 new cases and a comparison with the literature. Eur. J. Paediatr. Neurol..

[27] Crow Y.J., Leitch A., E Hayward B., Garner A., Parmar R., Griffith E., Ali M., Semple C., Aicardi J., Babul-Hirji R. (2006). Mutations in genes encoding ribonuclease H2 subunits cause Aicardi-Goutières syndrome and mimic congenital viral brain infection. Vol. Nat. Genet..

[28] Cerritelli S.M., Crouch R.J. (2009). Ribonuclease H: The enzymes in eukaryotes. Vol. FEBS J..

[29] Fang C., Wei X., Wei Y. (2016). Mitochondrial DNA in the regulation of innate immune responses. Protein Cell.

[30] Gambardella S., Limanaqi F., Ferese R., Biagioni F., Campopiano R., Centonze D., Fornai F. (2019). ccf-mtDNA as a Potential Link Between the Brain and Immune System in Neuro-Immunological Disorders. Front. Immunol..

[31] Gergely P., Grossman C., Niland B., Puskas F., Neupane H., Allam F., Banki K., Phillips P.E., Perl A. (2002). Mitochondrial hyperpolarization and ATP depletion in patients with systemic lupus erythematosus. Arthritis Rheum..

[32] Garau J., Sproviero D., Dragoni F., Piscianz E., Santonicola C., Tonduti D., Carelli S., Tesser A., Zuccotti G.V., Tommasini A. (2021). Hydroxychloroquine modulates immunological pathways activated by RNA: DNA hybrids in Aicardi-Goutières Syndrome patients carrying RNASEH2 mutations. Cell. Mol. Immunol..

[33] Islam M.T. (2017). Oxidative stress and mitochondrial dysfunction-linked neurodegenerative disorders. Neurol. Res..

[34] Nagy G., Barcza M., Gonchoroff N., Phillips P.E., Perl A. (2004). Nitric oxide-dependent mitochondrial biogenesis generates Ca2+ signaling profile of lupus T cells. J. Immunol..

[35] Pazmandi K., Agod Z., Kumar B.V., Szabo A., Fekete T., Sogor V., Veres A., Boldogh I., Rajnavolgyi E., Lanyi A. (2014). Oxidative modification enhances the immunostimulatory effects of extracellular mitochondrial DNA on plasmacytoid dendritic cells. Free Radic. Biol. Med..

[36] Guo R., Gu J., Zong S., Wu M., Yang M. (2018). Structure and mechanism of mitochondrial electron transport chain. Biomed. J..

[37] Gkirtzimanaki K., Kabrani E., Nikoleri D., Polyzos A., Blanas A., Sidiropoulos P., Makrigiannakis A., Bertsias G., Boumpas D.T., Verginis P. (2018). IFNα Impairs Autophagic Degradation of mtDNA Promoting Autoreactivity of SLE Monocytes in a STING-Dependent Fashion. Cell Rep..

[38] Signati L., Allevi R., Piccotti F., Albasini S., Villani L., Sevieri M., Bonizzi A., Corsi F., Mazzucchelli S. (2021). Ultrastructural analysis of breast cancer patient-derived organoids. Cancer Cell Int..

[39] Bronner D.N., O’Riordan M.X. (2016). Measurement of Mitochondrial DNA Release in Response to ER Stress. Bio. Protoc..

