# Supplementary material for: Characterization of Mitochondrial Alterations in Aicardi–Goutières Patients Mutated in RNASEH2A and RNASEH2B Genes
Source: Int J Mol Sci. 2022 Nov 21;23(22):14482. doi: 10.3390/ijms232214482 (PMC9692803; doi:10.3390/ijms232214482)
Supplement: Supplementary file 1 [file ijms-23-14482-s001.zip › ijms-2029000-supplementary.pdf]

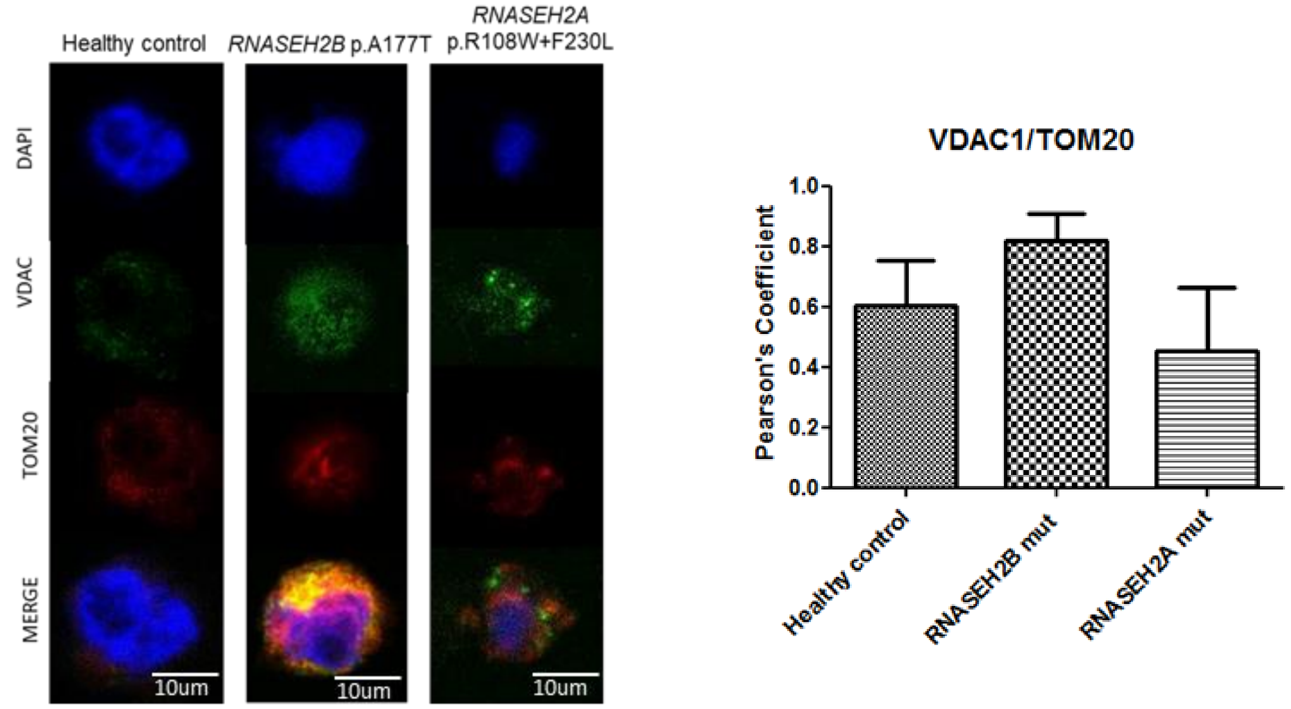

Figure S1. Fluorescence colocalization analysis (Pearson's coefficient) of LCLs derived from a healthy control and AGS patients mutated in *RNASEH2B* and *RNASEH2A* genes stained with VDAC1 antibody (green), TOM20 (red) and DAPI for nuclear staining (blue).

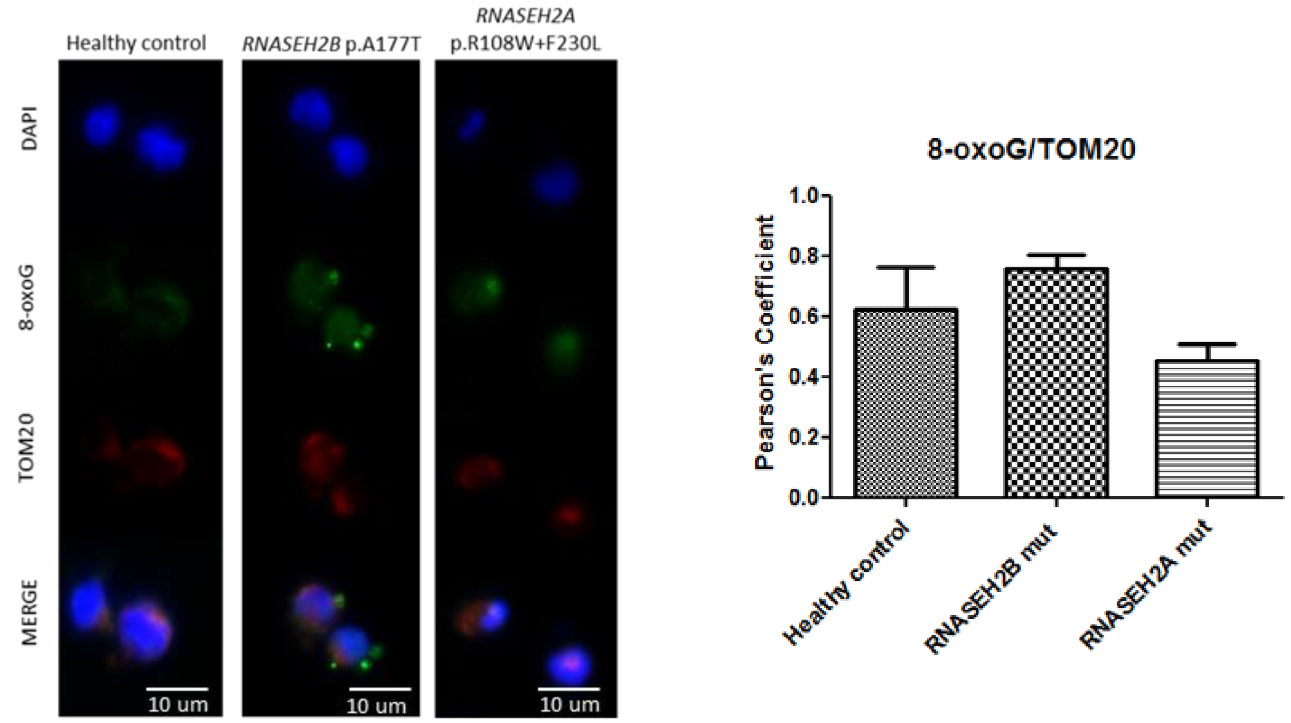

Figure S2. Fluorescence colocalization analysis (Pearson's coefficient) of LCLs derived from a healthy control and AGS patients mutated in *RNASEH2B* and *RNASEH2A* genes stained with 8-oxoG antibody (green), TOM20 (red) and DAPI for nuclear staining (blue).
